# Constipation in Infants

**Published:** 1882-03

**Authors:** 


### Constipation in Infants.

The following are some of the remedies found useful by Dr. D. H. Cullimore (*London Lancet*): 1. A pellet of butter and brown sugar or treacle every morning fasting, or a little raspberry jam. 2. The morning insertion into the rectum of a conical piece of white curd soap about two inches and a half long. It must be first dipped in warm water, held *in situ* for five minutes, and withdrawn. 3. Daily friction over the body, from the right iliac region along the course of the gut, with a little salad oil. In India I have used cocoanut oil advantageously. Cod-liver oil is very useful when its smell is not objected to. *En passant*, I may say that I have at present under my care a girl of fifteen who for a couple of months has suffered from obstinate constipation. She has lately had typhoid. Both mild and strong purgatives were ineffectual, and it has now yielded to cod-liver oil friction. Assiduous friction, without any unguent, is often equally useful. Patience, however, is necessary. A teaspoonful of fluid magnesia in the food is a good plan. Tomato jelly is sometimes used in India with benefit. Whatever plan may be adopted, it is well to supplement it with the internal administration of half a drop of tincture of nux vomica three times a day; a quarter of a drop is sometimes sufficient. Minute doses of sulphur also answer well.—*Louisville Medical News.*

